# Mixed germ cell tumor of sacrococcygeal region; A case report with literature review

**DOI:** 10.1016/j.amsu.2022.103247

**Published:** 2022-01-15

**Authors:** Wrya N. Sabr, Fahmi H. Kakamad, Abdulwahid M. Salih, Rawezh Q. Salih, Karzan M. Salih, Berwn A. Abdullah, Ahmed G. Hamasaeed

**Affiliations:** a Smart Health Tower, Madam Mitterrand Street, Sulaimani, Kurdistan, Iraq; b College of Medicine, University of Sulaimani, Kurdistan Region, Iraq; c Kscien Organization, Hamdi Str, Azadi Mall, Sulaimani, Kurdistan, Iraq; d Iraqi Board for Medical Specialties, Department of General Surgery, Sulaimani Center, Iraq; e Faculty of Medical Sciences, School of Pharmacy, University of Sulaimani, Sulaimani, Kurdistan, Iraq

**Keywords:** Germ cell tumor, Gonadal, Extragonadal, Yolk sac tumor, Sacrococcygeal area

## Abstract

**Introduction:**

Mixed germ cell tumors are uncommon tumors that contain two or more types of malignant, primitive, or germ cell components. This study aims to report a rare case of extragonadal mixed germ cell tumor of the sacrococcygeal area.

**Case report:**

A 4-year-old female presented with lower back swelling for two weeks. It was associated with pain and fever. Investigations showed elevated Alpha-fetoprotein and normal beta HCG levels. Magnetic resonance imaging showed a large well defined heterogeneous mass at the midline of the lower coccygeal region, which displaced the rectum anteriorly without invasion. Surgical dissection of the mass with excision of the coccyx was performed. Afterward, the patient was referred to an oncology center for chemotherapy.

**Discussion:**

The emergence of extragonadal germ cell tumors may be caused by a disruption in the migration of primordial germ cells along the urogenital ridge, which then undergo a malignant transformation as a consequence of their microenvironment. Another idea states that extragonadal germ cell tumors occur when germ cells that routinely migrate into the extragonadal region during embryogenesis undergo malignant transformation.

## Highlights

• Germ cell tumors (GCT) are rare and diverse group of heterogenous tumors.
• Young people's gonads (ovary or testes) are frequently affected.
• Mixed GCTs are uncommon tumors that contain two or more types.
• Mixed GCTs of the sacrococcygeal region are a rare occurrence.

## 1 Introduction

Germ cell tumors (GCTs) are a rare and diverse group of heterogeneous tumors that include benign and malignant histologies [1]. They arise from primordial germ cells and are categorized into two types: germinomatous and non-germinomatous types [2]. Young people's gonads (ovary or testes) are frequently affected. GCTs are described as extragonadal if there is no primary tumor in the testes or ovaries [3]. Extragonadal GCTs usually appear in the mid-axis of the body. In order of frequency, the anterior mediastinum, retroperitoneum, pineal, and suprasellar areas are the most common sites of occurrence in adults [3]. Mixed GCTs are uncommon tumors that contain two or more types of malignant, primitive, or germ cell components; they account for approximately 8% of all malignant GCTs. The most prevalent subtypes of GCTs are those with a combination of yolk sac tumor and dysgerminoma [4]. Mixed GCT of the sacrococcygeal area is a rare occurrence.

This study aims to report a rare case of extragonadal mixed GCT of the sacrococcygeal area in line with SCARE 2020 guidelines [5].

## 2 Case report

**Patient's information**: A 4-year-old female presented to the pediatric department with lower back swelling for two week duration. She was born with a normal vaginal delivery at 38 weeks of gestation and was not admitted to the neonatal intensive care unit. She was breastfed for two years and had no significant past medical or surgical histories during her upbringing. She was born from a non-consanguineous marriage, and there is no family history of a similar disease.

**Clinical findings**: The condition was associated with pain and fever. On digital rectal examination, a firm mass was palpable on the right upper anal sphincter.

**Diagnostic assessment**: Alpha-fetoprotein (AFP) was elevated (>1000), while beta HCG was normal. Complete blood count (CBC), sodium (Na), potassium (K), chloride (Cl), and serum creatinine all were within the normal reference ranges. Magnetic resonance imaging (MRI) showed a large well defined heterogeneous mass at the midline of the lower coccygeal region measuring (55*47mm) and slightly extended to the right side. The mass was encasing the lower two coccygeal segments and possibly invading the right puborectalis muscle. The mass was displacing the rectum anteriorly without invasion.

**Therapeutic intervention**: Surgical dissection of the mass under general anesthesia in a prone position was performed. Coccyx was excised, and the rectum was intact. The surgical specimen was sent for histopathological examination. The result showed a mixed germ cell tumor (70% embryonal carcinoma, 30% yolk sac tumor). The largest tumor diameter was 5.5 cm.

**Follow-up**: The postoperative course was uneventful. The patient was referred to an oncology center, and she received four cycles of chemotherapy. The last MRI reported no obvious residual or recurrent mass.

## 3 Discussion

Extragonadal GCTs are GCTs with histology linked with gonadal origin. Primary extragonadal GCTs are rare, and the actual incidence of this form of cancer is unclear. It is believed that they account for 3%–5% of all GCTs [6]. Mature and immature teratomas, seminomas, yolk sac tumors, embryonal carcinomas, choriocarcinomas, and mixed GCTs are among the pathological subgroups. Mixed GCTs are uncommon and consist of at least two malignant GCT components [7]. They typically appear in the body's mid-axis; 50–70% in the mediastinum, 30–40% in the retroperitoneal region [8]. The cause of extragonadal GCTs is uncertain; in rare cases, they have been linked to Klinefelter syndrome [9]. There are several ideas about the emergence of extragonadal GCTs. One theory is that they are caused by a disruption in the migration of primordial germ cells along the urogenital ridge, which then undergo a malignant transformation as a result of their microenvironment. Another idea states that extragonadal GCTs occur when germ cells that routinely migrate into the liver, bone marrow, and brain during embryogenesis undergo malignant transformation [10].

The clinical course is unpredictable, with a possibility of aggressive behavior and extensive metastases [11]. Sacrococcygeal germ-cell tumors are often identified antenatally on routine imaging and, if large enough, can cause hydrops fetalis or labor obstruction [12]. In neonates, it generally manifests as an external palpable lump; however, it can also manifest as discomfort while sitting, buttock asymmetry, or bladder, bowel, and lower limb dysfunction [13]. Any midline tumor should be evaluated for a germ-cell tumor diagnosis [12]. The current case presented with lower back swelling and was associated with pain and fever.

The most frequently used initial imaging technique is sonography, which is generally followed by MRI [14]. Primary extragonadal mixed GCTs have nonspecific computerized tomography (CT) performance; they are shown as heterogeneous tumors with regions of hemorrhage, necrosis, and heterogeneous enhancement. MRI, on the other hand, can define the mass and its connection to the surrounding structures, identify benign or malignant tumors, and detect lymph node and distant metastases, giving crucial data for tumor staging before treatment and surveillance following therapy [15]. The MRI of the present case showed a large well defined heterogeneous mass in the lower coccygeal region with slight extension to the right side with the invasion of the right puborectalis muscle. The serum AFP level, morphology, and immunohistochemistry staining for C-kit, placental alkaline phosphatase, AE1/AE3, and CD30 all strongly suggest the diagnosis of mixed GCT [16]. Depending on the components of the tumor, mixed GCTs produce AFP and beta HCG [7]. AFP of the current case was elevated (>1000), while beta HCG was normal. A biopsy is necessary for a definite diagnosis and treatment of extragonadal GCTs; the majority of the patients show strong evidence of germ cell characteristics or teratoma, while a minority could present with a poorly differentiated tumor lacking distinct germ cells features [3]. Until a primary testicular tumor is ruled out, extra-gonadal GCTs are regarded as metastases from occult or "burned out" gonadal malignancy. To rule out a testicular tumor, palpation of the testicles is inadequate. An ultrasound should always be conducted, and gonadal biopsy is debatable but not advised [9]. Teratoma is the most common benign histology of sacrococcygeal GCTs, and the most common malignant histology is the yolk sac tumor, which is frequently admixed with teratoma [17]. The histopathology of the current case showed a mixture of embryonal carcinoma and yolk sac tumor.

The treatment of mixed GCTs concentrates on the most malignant components. Complete surgical excision is the primary therapy for children with endodermal sinus tumors with adjuvant chemotherapy assessed on a specific instance basis [7]. The platinum-based PEB (cisplatin, 90 mg/m2 day 1; etoposide, 120 mg/m2 days 1–3; bleomycin, 15 mg/m2 day 2, administered every 21 days) regimen was the first-line therapy utilized in the treatment of GCT [18]. 5%–15% of these patients will have persistent marker increase or disease progression; therefore, they will be treated with second-line chemotherapy with ifosfamide and cisplatin with either etoposide (VIP) or vinblastine (VeIP) [19]. VAC (vincristine, actinomycin D, cyclophosphamide) chemotherapies can be used as neoadjuvant therapies in some cases [18]. Adjuvant chemotherapy given after surgery has improved the survival rate of GCT patients [7]. If tumor biomarkers return to normal but there is a residual lump larger than 1cm, surgery should be undertaken [29]. If a viable carcinoma is found, two further rounds of chemotherapy should be administered [20]. In the current case, the tumor was dissected and the coccyx was excised, and the patient was sent for chemotherapy.

Mixed GCTs, which mostly consist of teratomas with other malignant components (other germ cell tumors, carcinomas, or sarcomas), have been observed to be more aggressive. More than half of affected individuals die within two years due to local invasion or distant metastases (lymph nodes, liver, lung, heart, bone, and brain) [21]. Patients who relapse after initial treatment for extragonadal GCTs have a very bad prognosis, perhaps worse than patients with primary gonadal non-seminomatous testicular cancer [6]. The majority of the relapses occur within a year after the diagnosis. Thus, a careful follow-up with serial AFP level monitoring should be performed for at least two years [22].

In conclusion, the occurrence of primary mixed GCT in the sacrococcygeal region is a rare condition. Proper initial surgery with proper staging biopsies, followed by combination chemotherapy, can significantly improve these patients' prognosis.

## Ethical approval

The manuscript approved by ethical committee of the University of Sulaimani.

## Sources of funding

No source to be stated.

## Author contribution

Abdulwahid M. Salh: major contribution of the idea, literature review, final approval of the manuscript.

Wrya N. Sabr: Surgeon performing the operation, final approval of the manuscript.

Fahmi H. Kakamad, Karzan M. Salih: literature review, writing the manuscript, final approval of the manuscript.

Rawezh Q. Salih, Berwn A. Abdullah, Ahmed G. Hamasaeed: literature review, final approval of the manuscript.

## Registration of research studies

1. The study was not registered because it is a case report.

## Guarantor

Fahmi Hussein Kakamad is the Guarantor of this submission.

## Consent

Written informed consent was obtained from the family of the patient for publication of this case report and accompanying images. A copy of the written consent is available for review by the Editor-in-Chief of this journal on request.

## Provenance and peer review

Not commissioned, externally peer reviewed.

## Declaration of competing interest

There is no conflict to be declared.
